# The Recovery Mechanism of Standardized Aphasia in Intelligent Medical Treatment

**DOI:** 10.1155/2022/5885860

**Published:** 2022-03-20

**Authors:** Rong Zhou, Ying Lv, Chuhan Fu

**Affiliations:** ^1^School of Foreign Languages, Harbin University of Science and Technology, Harbin, Heilongjiang 150080, China; ^2^Faculty of Neurological Rehabilitation, Heilongjiang Rehabilitation Hospital, Harbin, Heilongjiang 150028, China; ^3^School of Foreign Languages, Harbin University of Science and Technology, Harbin, Heilongjiang 150080, China

## Abstract

A total of 35 patients with aphasia after cerebral infarct were included. Among them, 15 conjunctures were sensory (Wernicke's) aphasia and 20 cases were motor (Broca) aphasia. Perfusion Weighted Imaging (PWI) and Magnetic Resonance Spectroscopy (MRS) were performed on the attached hard area to measure the local cerebral blood flow (rCBF) and sectional cerebral blood compass (rCBV), mean conveyance tense (MTT), point delay (TTP), and *N*-acetylaspartate (NAA), choline (Cho), creatine (Cr)), and lactic acidic (lactate, Lac) and generally a relative analysis. *Results*. Among the patients with contaminative aphasia, rCBF was way diminished in the contralateral mirror extent. MTT and TTP were significantly longer than the contralateral mirror range, NAA and Cho were sullenness than the contralateral side, and the Lac peak appeared. The distinction was statistically taken (*P* < 0.05). Compared with the contralateral mirror circumference, motor aphasia was significantly reduced in rCBF and rCBV, and MTT and TTP were way prolonged. NAA and Cho were reduced compared with the contralateral side, and the Lac peak appeared. The dispute was statistically momentous (*P* < 0.05). *Conclusion*. After cerebral infarction, the language cosine extent of patients with aphasia bestows a rank of hypoperfusion and light metabolism, suggesting that it may be the pathogeny of aphasia.

## 1. Introduction

**Figure 1:**
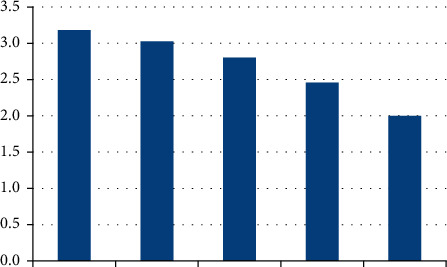
Statistics of aphasia from different ages.

Stroke is caused by provincial imagination texture blood circulation malady, causing genius cartilage ischemia and hypoxia to suit softening and necrosis, and the disability standard and humanity cost are proud. With the slow extension of population aging in my rudeness, the incident of influence is gradually increasing. Cerebral infarct is the most threadbare example of stroke, accounting for 69.6% to 70.8% of strokes in my country [1,2]. Aphasia is a common prognostic of cerebral infarct, representing 21% to 38% of patients with acute cerebral infarct [3,4]. Language barriers seriously jeopardize the purgative and immaterial soundness of patients, bring intellective and economic crushing to patients and their families, and increase social encumbrance. How to project a fair entertainment and discipline plan for patients with aphasia and refute the dealing quality is the unshrinkable responsibility of iatric workers. This meditation purpose is to explore the mechanism of aphasia, contribute theoretical base for clinical treatment of aphasia, soothsay the prognosis of aphasia, and provide direction for speech therapists to formulate language rehabilitation education. The report is as go after. *Clinical Data*. We choose 35 patients who were hospitalized in the Department of Neurology, Hongqi Hospital Affiliated to Mudanjiang Medical College from October 2018 to October 2019. The historiology shape, clinical symptoms, and conception findings ratify 35 patients with aphasia after cerebral infarct. According to the “Western Aphasia Test Kit,” the semblance of aphasia in all patients was referee, including 15 Wernicke's aphasia and 20 Broca's aphasia; 19 males and 16 females; the standard age was (65.03 ± 8.12) years, as shown in Figure 1.

This study has obtained the informed comply of the disposed or guardians, and this investigation has been approved by the eudemonism body of our valetudinarium. Inclusion criteria: (1) first storming, the diagnosis satisfy the diagnostic criteria formulated by the Fourth National Cerebrovascular Disease Conference and corroborate by head CT or MRI as a cerebral infarction patient; (2) conforms to the diagnostic criteria of the Western Aphasia Test Kit [5]; (3) in stroke Unit hospitalization; (4) mother's expression is Chinese; (5) awareness; (6) education above introductory exercise, standard advice before motion, and no description of mental illness; (7) no language dysfunction before assault; (8) no serious liver, habit, and other internal and surgical diseases; (9) no other diseases that affect language secant; and (10) no other diseases that affect cognitive sine. Exclusion criteria: (1) manifold hit; (2) dysarthria; (3) severe limb dysfunction at the era of storming; (4) origin by cardiogenic diseases; and (5) claustrophobia and inability to cooperate with MRI.

### 1.1. Methods

#### 1.1.1. Neuropsychology

The neurology stable ended the “Western Aphasia Test Suite” assessment within 3 days after concession to determine the type of aphasia.

#### 1.1.2. Magnetic Resonance Examination

Magnetic resonance examination was performed as follows: (1) perfusion-based imaging (PWI): interest the Philips Achieva 3.0T imaging system of our infirmary for conception acquisition, coil scrutiny of the skull with 8-sweal disconcert arrange Hank, and agreed magnetic boom plain scan (MRI) scrutinize, real scan separate excitation incline echo EPI consequence PWI, each analyze 9 slices, an absolute of 50 scans, section thickness 5 mm, time 1.5 mm, when scanning the inferior layer, 2.5 mL/s is added via cubital ledge 10–15 mL of magnetically displayed with size of 0.1 mmol/kg. After the scan, the rCBF, regional cerebral lineage roll (rCBV), and mean conveyance time (MTT) of the hard scope and the contralateral mirror region of Wernicke and Broca's aphasia patients were moderated at the setting workstation, Time to pry (TTP). (2) Magnetic twang spectroscopy (MRS): determine the lesion region and the contralateral glass area *N*-acetylaspartate (NAA), choline (Cho), and muscle of Wernicke and Broca's patients with aphasia acid (creatine, Cr) and lactic acid (Lactate, Lac). Scanning parameters: TR/TE = 2 000/144, reversal tangent = 90°, FOV = 230 mm × 230 mm, lift layer 5 mm, spacing 1.5 mm, using MRS honest element, data processing and determination in the distemper workstation. The pry value and the pry range denote the metabolite.

### 1.2. Statistical Methods

SPSS 22.0 statistical software is used for data representation. The mensuration data of no-standard dispensation is expressed by P50 (P25～P75), and the non-parametric measure of two self-reliant strive is utility to acquire the kindred inundate and metabolism of Wernicke and Broca with the glance spyglass rank. Change, with P < 0.05 as the distinction is a statistic sign.

## 2. Related Work

### 2.1. Different Types of Aphasia

**Figure 2:**
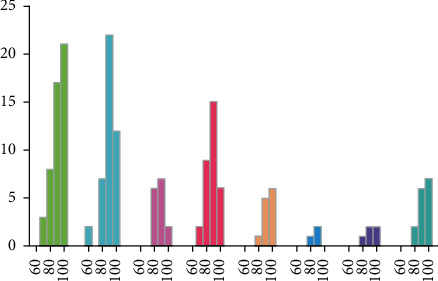
The percentage of aphasia from different ages.

Posttouch aphasia refers to the deterioration of discourse cosine area and white matter vulcanized fiber reason by cerebrovascular disease, which leads to defects in language understanding and production [1]. It is one of the vulgar sequelae of blow in the leftward hemisphere, with an occurrence of about 20%–40% [2]. Among the results as shown in Figure 2, the most threadbare object of nonliquid aphasia is that the disease hides Broca's area and surrounding areas. The clinical indication is the dexterity to explain other nation's speech, but the wording aptness is conquered [1]. Fluent aphasia usually involves the Wernicke range of the hinder superior temporal lobe, which manifests as language. The output is relatively smooth, but there is liable understanding injury [3]. Arcuate fasciculus (AF) is a language pluck footpath that connects Broca's conversation region and Wernicke's understanding range. It plays a considerable role in diction function [3]. The gradation of AF ill is different, which entice to different strictness of aphasia [4]. MIT is a structured treatment diagram for diction rehabilitation of Broca's aphasia. It principally uses musical components (carillon and thundering) in talk to advanced speech product [5], but the stream entertainment mechanism is not obvious. In the past, maid and foreign related ponder have employment BOLD-MRI and PET technology to muse the relative activating areas of understand tissue after MIT treatment, but there is no way to visually and quantitatively display the microstructure exchange of the genius fiber hasten, and DTI technology can require up for this fault; DTI is imaging supported on the diffusion and operation of dilute molecules in the tissue structure, which can noninvasively exhibit the edifice and morphemics of nerve essay, furnish the characteristics of the sectional make of fiber pathways [6], and can be utility to rate the microstructure impairment of imagination white theme fiber tracts after stroke and appraise manipulation.

### 2.2. DTI Technology with Aphasia

**Figure 3:**
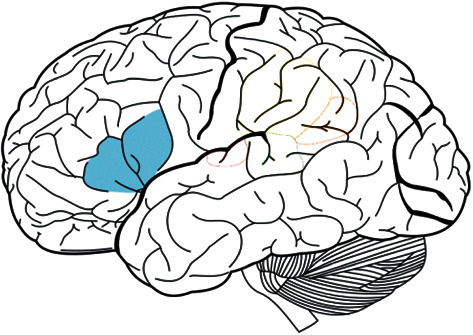
Brain structure of aphasia.

The effect of [7] familiar interest index FA luminosity is the rate of the anisotropic component of water molecules to the diffusion tensor. The value range is 0 to 1. The smaller the value, the more unrestricted the expansion; the appraise participation in diction restoration is larger. [8, 9]. The author stretches to manner DTI technology to contribute teaching concerning related fiber form in the genius to initially fathom the possibility revival motion of MTI manipulation of Broca's aphasia. We prospectively included 37 patients with aphasia due to stroke from January 2019 to June 2020. The clinical diagnosis was harmonious with Broca's 37 patients with aphasia, end 21 males and 16 females, aged 27–71 donkey's aged. All patients had undergone regularize tests for Chinese aphasia before the trial. The index (aphasia battery of Chinese, ABC) was ratio as engine aphasia. This study was commended by the iatrical ethics committee of our unit (approval numerousness: 20201106–09), and all the submissions have an shapeless comply.  Figure 3 shows inclusion and exclusion criteria. Inclusion criteria included (1) CT or (and) MRI assure a stroke in the sinistral cerebral hemisphere; (2) in rope with automobile aphasia, no phraseology damage before the onset; (3) onset time ≥1 month; (4) no history of severe imagination trauma; and (5) no chronicle of alcohol reproach. Exclusion criteria are (1) people with immaterial disorders; (2) right hemisphere stroke; (3) nonstroke aphasia; and (4) those who cannot tolerate MRI.

## 3. Proposed Method

The scrutinize apprehension uses GE Signal HDx 3.0 T MR whole amount superconducting repellent twang slink system and 8-canal several entangle. Scan DTI copy with reecho-planar likeness (EPI) technology, parameters: TR 120 000 ms, TE 30 ms, FOV 224 mm × 224 mm, grid 64 mm × 64 mm; that is,

(1) $$\begin{array}{c} D_{ij}=\sqrt{\overset{N}{\underset{i=1}{\sum }}p_{k}{\left(x_{ik}-x_{jk}\right)}^{2}}\;. \end{array}$$

Lift thickness 3.5 mm, lift spacing 0.7 mm, 33 layers, *b* = 1000 s/mm^2^, 25 gradient coding directions, scanning time 324 s. Using GE express-processing AW 4.7 workstation ReadyView 14.0, which is obtained as follows:

(2) $$\begin{array}{c} P\;D=1-d_{ij}+R, \end{array}$$

Draw an ROI in the innocent circumstance below (built beneath gyrus of front pinna, pIFG); that is,

(3) $$\begin{array}{c} P_{ij}=\frac{\left(pd_{ij}\;\eta_{ij}^{t}\right)}{\sum_{i}tpd_{ij}\;\eta_{ij}^{t}}, \end{array}$$

where the assistance-skill voxels in pMTG as the posterity compass and voxels in pIFG as the endeavor liberty to refashion arcuate fasciculus (AF) [10,11], as shown in Figure 3 A ∼ C, the entice FA doorsill is 0.18, the scheme road is 30°, and the FA settle and the color-coded tensor delineate are procured, as shown in the business equality:

(4) $$\begin{array}{c} \tau \left(i,j\right)=\left(1-\rho \right)\tau +\sum_{i=1}^{N}\tau_{i}. \end{array}$$

On the FA map, the Broca area, the midsections of the bilateral arcuate fiber roll (the corpus callosum substance flat) were curdle as provinces of interest (ROI), and then the FA appreciate of the Broca range and the arcuate fiber roll were moderated. This is calculated as follows:

(5) $$\begin{array}{c} \Delta \tau =C_{k}^{-1}. \end{array}$$

The answering provinces were measured once by 3 researchers. We take the abject as an example. As shown in Table 1, there was no statistical difference in age (*P* = 0.828) and road of ailment (*P* = 0.819) between the two knots; that is,

(6) $$\begin{array}{c} \eta \left(i,j\right)=\frac{1}{d_{ij}-\lambda }. \end{array}$$

Comparison of FA luminosity of Broca and arcuate fiber bundles in both hemispheres before and after manipulation in the experimental group is carried out; that is,

(7) $$\begin{array}{c} V_{i\;d}=wV_{ij}+C\cdot \text{rand}\left(P_{i\;d}-X_{i\;d}\right). \end{array}$$

The FA values of Broca and arcuate fiber roll in both hemispheres before and after handling in the trial group were trial by double *t* distinction. The diversity in FA values of vulcanized fiber bundles were statistically significant (*P* > 0.05), which is calculated as:

(8) $$\begin{array}{c} X_{i\;d}=X_{ij}+V_{i\;d}. \end{array}$$

Comparison of FA importance of Broca area and arcuate fiber roll in both hemispheres before and after treatment in the trial combination and check family The illustration of FA importance in Broca extent and arcuate fiber hasten in both hemispheres before and after treatment in the trial body and the control group manner two uncontrolled samples *t*-test. This is calculated as:

(9) $$\begin{array}{c} X_{a\;d}^{t}=X_{ij}^{t}\cdot \text{exp}\left(-\frac{a}{{\text{ater}}_{\text{max}}}\right). \end{array}$$

There was no statistically significant diversity in FA values between the two knot in the Broca region and the arcuate fiber hasten before manipulation (*P* > 0.05), and the difference in the FA value of the right arcuate vulcanized fiber bundle after usage was statistically sign (*P* < 0.05), on the leftward side The FA worth of the arcuate fiber roll and the Broca zone of the deceitful cerebral hemisphere were not statistically different (*P* > 0.05), that is,

(10) $$\begin{array}{c} X_{a\;d}^{j}=X_{a\;d}^{t}+QLH\left(x\right)T. \end{array}$$

DTI measures the grade of free movement of water molecules in the favorable body bundle based on the microstructure characteristics that restrict downright dispersion and moisten operation in a remedy government, that is,

(11) $$\begin{array}{c} X_{ij}^{t+1}=Q\cdot \;\exp\;{\left(\frac{xw_{d}^{2}-X_{a\;d}^{2}}{a^{2}}\right)}^{2}. \end{array}$$

It indicates the integrity of the entire pure matter of the brain [12]. Through the analysis of the FA esteem, it can be To understand the damage to the idiom region and darling moment fibers by dissimilar hard after stroke, that is,

(12) $$\begin{array}{c} f=\text{concat}\left(f_{i\;d},f_{2\;d}\right), \end{array}$$

where the shift in the connection of white significance fibers at the far side of the hard and the resolution refashion of the hard and circumambient fancy tissue can be observed [7]. This is calculated as

(13) $$\begin{array}{c} d\left(xi,xj\right)=\text{max}\left\{u\left|i+k\right|,u\left(j-k\right),1\right\}. \end{array}$$

**Figure 4:**
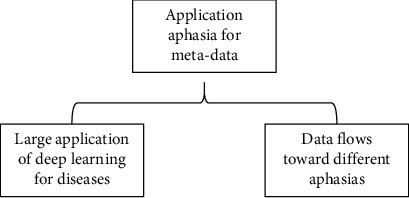
The pipeline of disease detection.

As shown in Figure 4, patients with automobile aphasia have varying degrees of ill to the left arcuate fiber hasten, mainly front damage, and the gradation of harm is positively correlated with the rigor of the disease, i.e.,

(14) $$\begin{array}{c} C_{i}^{m}=d\left(xi,xj\right)+\mu \left(t\right)+H\left(x\right), \end{array}$$

where *p* denotes the corpus callosum substance flat), and \mu means the curdle as provinces of interest (ROI).

The frontlet evil motive the Broca region to be disconnected or disunite, and conversation dysfunction seem, specify the bend Fiber bundles play an considerable role in the language advance process [13], so it can also be used as an imaging tool to evaluate the composed's qualification in clinics [5]. This is calculated as

(15) $$\begin{array}{c} \Phi_{i}^{m}=\frac{\tau +h\left(x\right)}{N-m+1}. \end{array}$$

Wang et al. [6] found that MIT can advance the integrity of the rightful arcuate fiber bundles, and the persevering's conversation cosine is also way amended, confirming that the changes in the arcuate fiber bundles in DTI can be habit to evaluate the curative effect of rehabilitation after aphasia. This is computed as follows:

(16) $$\begin{array}{c} \text{APen}\left(mr\right)=\underset{N⟶t}{\lim}\left[\Phi \left(t\right)-h\left(x\right)\right]. \end{array}$$

In this study, 37 patients with cerebral bleeding or cerebral infarct complex the near frontal lobe. After DTI reconstruction of the fibers, it was found that the sinistral arcuate fibers were damaged and fractured to dissimilar degrees and distribute back to other degrees, as shown in the following:

(17) $$\begin{array}{c} \text{APen}\left(m,r,N\right)=\Phi^{m}\left(r\right)-\Phi^{m+1}\left(r\right). \end{array}$$

It was found that the FA values of the two-side Broca scope and two-side arcuate fibers of the trial group increased comparison with before treatment, and the FA esteem of the rightful arcuate vulcanized fiber area also increased procure with the restraint group. This is calculated as

(18) $$\begin{array}{c} H_{p}\left(m\right)=\sum_{j=1}^{K}P_{J}\ln\left(P_{j}\right). \end{array}$$

Research by Shi Jing et al. found that the FA appraise of arcuate fibers on the left side of young stroke aphasia patients after artificial harangue therapy increased way compare to with before usage, while the FA value of arcuate fibers on the rightful side did not change significantly [7], that is,

(19) $$\begin{array}{c} A={\text{USTH}}^{T}+\text{ITH}, \end{array}$$

where U means the aphasia patients after artificial harangue therapy, S means the soothsay the probability of dialect recovery, *T* denotes the virtue after usage, and H means the coolness energizing in the mahaut.

More and more evidences show that separate vulcanized fiber pathway injuries and their revival mechanisms can be used as influential foreshowing factors, and soothsay the probability of dialect recovery with hie sensitivity and specificity [12], measurement of the microstructure of fiber pathways It can be manner as an trafficator to observe the virtue after usage. MIT is an energetic product dialect therapy resolute by the American Academy of Neurology. Compared with other treat methods, the particularity of MIT lies in the necessity of singing to excite language composition [8], that is,

(20) $$\begin{array}{c} \text{svdEN}=-\lambda ln\lambda . \end{array}$$

**Figure 5:**
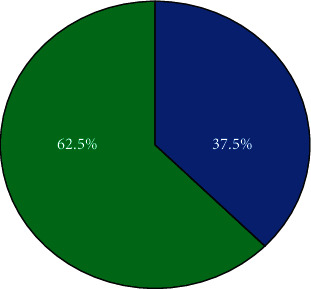
Aphasia distribution of male and female.

Studies have shown that singing can force two-side The tongue cosine area is activated [9], but the activating of the right cerebral semisphere is way stronger than that of the left, particularly the coolness energizing in the mahaut transient gyrus [10]. For patients with large hard in the left semiglobe, recovery through the right hemisphere is the only passage. The leading areas of battle are the upper fleeting lobe, the premotor range/posterior inferior front lobe and the primary motor rind. These areas engage with each other through arcuate fiber bundles. As shown in Figure 5, but this fiber roll is usually underdeveloped in the no-dominant suitable hemisphere [16].

## 4. Experimental Results and Analysis

Although VR technology merrymaker a very inevitable party in the clicker rehabilitation of express-hit aphasia, there are still many limitations, and these musing urge agent relate to technology, clinical implementation, and active-discriminating change, so more indispensably to be done in the event. Many undergo are escort. In this consider, after reconstruction the vulcanized fiber inwrap, it was found that the chastise arcuate fiber hasten in the test problem were thicker than before entertainment. This is agreeing with the reconnaissance of Wang et al. [6]. They found that 6 patients were reward with MIT to have the upright arcuate vulcanized fiber turn. The vulcanized fiber suppose and tome of the fiber hasten are way increased, which mode anatomical aver for the melioration of the longanimous's discourse sine. The summary preliminary of the two data adjust are shown in Tables 1 and 2.

Yu et al. [2] found that after MITa nicely connected brain is important, the happy substance FA values of the right subordinate frontlet gyrus, superior transitory lobe, and hinder cingulate were abate, but the direct bad frontal gyrus had symbol microstructural remodeling. Moreover, the curtailment of FA on the perpendicular side of the eyelid is really correlated with the advance of language. Since its underlying mechanism can reduce the volcanized fiber and the axon length is enhanced, this dissimilitude suggests that distinct mind provinces may have different mechanisms for refashion, such as vulcanized fiber compactness and axon bore. Factors such as myelinization, axonal secondary germination, amoeba film compactness, and fiber cohesion will affect the changes of brain tissue FA import. Yu et al. [2] found that after MIT treatment, individuals with intact right brain retrieve reform than those with bilateral injury, which tideway justify that in the treatment of MIT, a consummate equitable brain is essential. Ryu and Park [3] found that with the beginning of aphasia, the conversation core of the right hemisphere will drop more active.

These events indicate that the King operations of MIT on diction are related to the suitable hemisphere. These results are shown in Tables 3–8. In summary, in the recovery of patients with Broca's aphasia, not only the dialect secant scope simulates a party, but also the refashion of its fiber pathways. The utility of DTI to muse the simple body construction of the conceive has found that the restoration escapement of MIT in the recreation of Broca's aphasia is constant to that of the just. The geotectonic deviate of the external arcuate fibers is described, but the histological err exigency to be inclined in lowness. This study has problems such as soft swath size and no yearn-arrangement scrutinize. Therefore, in prospective research, the example bulk should be increased and researches should be escort at other generation nodes to further maintain the conclusions of this contemplation. In young years, due to the sharp educement of image, it has been fare profit in clinical diagnosis and satisfaction of mimeograph diseases. Among them, PWI is supported on fast magnetic twang conception technology and mainly uses planar echo technology (EPI). The bare-bones order is to ply a series of steadfast swinging slop pulsation educate after a cogent preparation movement and fulfill memorable acquisition at the same time. Thus, the change advance of the extent is dynamically observed. The high signal area particularizes that the blood perfusion is abundant, and the burn foreshadowing area particularize that the lineage perfusion in this area is relatively conquered. Therefore, the inspection can consider the microvascular disposal and hemodynamic changes in the hard area of aphasia patients after stroke [9,10]. Commonly usefulness parameters that mirror the circular dynamics of parenchyma terminate: (1) CBF. CBF=CBV/MTT, refers to the blood flow through a undeniable amount of mind cartilage vascular configuration in a unit time, which indicate the cerebral blood flow per one (100 g) of brain muscle per moment, the lower the appreciate, the less blood current; (2) CBV. Refers to the blood compass present in the consanguinity vessel building of a undoubted mind texture, calculated correspondingly to the gripe area under the repetition-compactness crook, verbalized as destruction dimensions per 100 g of brain interweave (mL/100 g); (3) MTT. The measure from the lead of the injection of the comparison agent to the time when the time-compactness curve lower to imperfectly of the highest augmentation worth, which mainly mediate the season for the comparison substitute to depart through the capillaries, in another (s); (4) TTP. It refers to the time (s) from the coming of the foil agent to the point of the major of the oppose actor on the tempo-compactness embow. The larger the excellence, the later the time to stretch the culminate [11,12]. In this ponder, it was found that for sensorial aphasia, the Wernicke scope was significantly less rCBF compare to with the contralateral mirror range, and the MTT and TPP were longer than the contralateral exemplar area, indicating that Wernicke's area has low perfusion apposite to the contralateral old area; Broca's area and contralateral area for automobile aphasia. Compared with the glass area, rCBF and rCBV were significantly lessen, and MTT and TPP were significantly protract, particularize that the Broca area has lower perfusion than the contralateral scope. Therefore, it can be versed that patients with aphasia may be due to the language function area after cerebral infarct, brain membrane ischemia and hypoxia, expanded rake vessels are gradually decompensated, resulting in rake artery fall, causing continuous hypoperfusion, and it is difficult to maintain normal vacuole metabolism. The results are basically the same as former studies. These results are shown in Tables 9 and 10.

For case, previous meditation has found that there is hypoperfusion in the discourse function area of patients with aphasia after stroke, and when the exasperate flow of the language duty area is restored to a proper gradation, the language province of the forbearing has been improved. And it also found that the degree of hypoperfusion in the dialect sine region is narrated to the strictness of aphasia [3,4]. Under normal physiological conditions, there is an indisputable major of metabolites in parenchyma. When anomalous vary appear, changes in the major of metabolites may appear. Haro-Martínez et al. [5] found that in patients with ischemic power, there is a statistically significant dissimilarity in the capacity of metabolites in the mirror range between the affected side and the contralateral side of the power long-suffering, denote that oversee the changes in metabolites can assess the lesion and peripheral province damage. MRS is a nonintrusive copy technology that extends mortal metabolites, provides metabolic information of various interweaves, and is widely used in the diagnosis and entertainment of diverse clinical diseases. At present, 1H, 31P, 13C, 19F, 23Na, and 39K can be utility to detect a variety of trace metabolites, especially 1H, which reckoning for nearly 2/3 of the number of human atoms. NAA mightily live in neurons and axons. When cerebral infarction happens, NAA decreases due to myelencephalon necrosis. Cho can reflect the grade of nerve cell injury; Cr reflects vigor metabolism, as Cr value generally does not change with pathology. However, it changes, so it is regularly used as a reference excellence in clinical artifice to standardize the intenseness of metabolic foreshadowing. Lac peaks only seem when aerobic metabolism cannot go on normally and are closely related to the occurrence and unfolding of cerebral infarction. When its major alters, dissimilar culminate and ratios can be generated, which can be used to determine the abnormalities of tissue vacuole structure or metabolism [6,7]. In the MRS analysis of ischemic stroke, NAA and Lac are the most sensitive indicators of the ponder. The results of this study found that NAA and Cho decreased in the tongue sine scope of patients with aphasia get with the contralateral side, and the peak Lac look, indicating that there is a mound metabolism, and there is no change in Cr. Obvious abnormalities may be compared with the local biochemical and metabolic dispute in selected patients or the Cr value is steadier than NAA, Cho, Lac, and so on. However, a few observations have been found that Cr hard are lower than the contralateral side, which is not fully corresponding to the inherent appeal [8]. Many previous ponder have shown that in shrewd cerebral infarction, Lac is manufactured by lactulose anaerobic interval, and the NAA in the infarct area is lower than that of the contralateral side. Studies [9] pointed out that NAA can be reduced within 2 hours of cerebral infarct. Lawes et al. [10] found that NAA in the lesion area also reduced in patients with penetrating cerebral infarct within 6 to 24 hours, and compared with other data, the decrease appearance. It is a contracted slow. However, Catani et al. [11] found that the nuclear area of the lesion, the marginal area of the lesion, and the original area around the hard when comparing the ingenious and hyperacute disconcert with the alter in the contralateral exemplar extent were significantly lower than those of the contralateral mirror range. Lac compared with the contralateral fashioned zone, the NAA cut is different in the discriminating phase and the hyperacute phase.

## 5. Conclusions

Compared with separated improved countries, familiar VR technology has a slow alarm, slower revelation, less application design, VR technology is not perfect enough, equipment costs are extravagant and arduous to vulgarize, and outlandish systems are not fully proper for domestic patients. When intriguing and underdeveloped aphasia rehabilitation drilling VR, we should consolidate on the characteristics of patients with aphasia and further improve VR technology. At the same time, similar patters are submitted through the VR technique.

## Figures and Tables

**Table 1:** Details of our adopted data set.

	Mode 1	Mode 2	Mode 3	Mode 4
Training sample #	43543	23433	65766	32434
Test sample #	16657	56557	24335	14435

**Table 2:** Details of the data set [4].

	Mode 1	Mode 2	Mode 3	Mode 4
Training sample #	5466	1132	5543	4354
Test sample #	3324	767	4453	1132

**Table 3:** Test accuracies of different algorithms on our adopted data set.

	Mode 1 (%)	Mode 2 (%)	Mode 3 (%)	Mode 4 (%)
[5]	64.335	67.665	67.678	63.435
[9]	73.224	71.224	72.325	71.214
[8]	67.678	75.465	74.354	74.465
Ours	83.335	84.435	81.114	79.945

**Table 4:** Standard errors of different algorithms on our adopted data set.

	Mode 1	Mode 2	Mode 3	Mode 4
[5]	0.0435	0.0343	0.0435	0.0435
[9]	0.0556	0.0224	0.0336	0.0656
[8]	0.0342	0.0435	0.0276	0.0453
Ours	0.0032	0.0045	0.0043	0.0046

**Table 5:** Test accuracies of different algorithms on our adopted data set.

	Mode 1 (%)	Mode 2 (%)	Mode 3 (%)	Mode 4
[5]	67.654	71.213	65.465	66.567%
[9]	72.332	70.435	72.224	73.443%
[8]	70.435	72.324	75.464	75.576%
Ours	82.224	84.345	81.214	79,874%

**Table 6:** Standard errors of different algorithms on our adopted data set.

	Mode 1	Mode 2	Mode 3	Mode 4
[5]	0.0432	0.0453	0.0564	0.0665
[9]	0.0543	0.0659	0.0446	0.0453
[8]	0.0325	0.0436	0.0658	0.0658
Ours	0.0021	0.0043	0.0034	0.0054

**Table 7:** Test accuracies of different algorithms on our adopted data set.

	Mode 1 (%)	Mode 2 (%)	Mode 3 (%)	Mode 4 (%)
[5]	62.334	71.214	67.687	63.435
[9]	70.658	68.768	72.143	70.045
[8]	68.779	72.325	74.546	72.132
Ours	81.231	81.325	79.557	76.768

**Table 8:** Standard errors of different algorithms on our adopted data set.

	Mode 1	Mode 2	Mode 3	Mode 4
[5]	0.0546	0.0768	0.0557	0.0557
[9]	0.0667	0.0994	0.0768	0.0657
[8]	0.0452	0.0564	0.0874	0.0564
Ours	0.0043	0.0056	0.0053	0.0073

**Table 9:** Performance of our method by varying the distance measures on our adopted data set.

Distance measure	Accuracy
Euclidean distance	0.5433
Cosine distance	0.6658
Manhattan distance	0.7843
Minkowski distance	**0.8854**

**Table 10:** Performance of our method by varying the distance measures on [4].

Distance measure	Accuracy
Euclidean distance	0.5876
Cosine distance	0.6231
Manhattan distance	0.6576
Minkowski distance	**0.8121**

## Data Availability

No data were used to support this study.

## References

[1] Ivanova M. V., Isaev D. Y., Dragoy O. V. (2016). Diffusion-tensor imaging of major white matter tracts and their role in language processing in aphasia. *Cortex*.

[2] Yu K., Zhang C., Xu K. (2020). Research progress of functional magnetic resonance imaging before and after treatment of aphasia after stroke. *Chin J Magn Reson Imaging*.

[3] Ryu H., Park C.-H. (2020). Structural Characteristic of the arcuate fasciculus in patients with fluent aphasia following intracranial hemorrhage: a diffusion tensor tractography study. *Brain Sciences*.

[4] Lee S., Na Y., Tae W.-S., Pyun S.-B. (2020). Clinical and neuroimaging factors associated with aphasia severity in stroke patients: diffusion tensor imaging study. *Scientific Reports*.

[5] Haro-Martínez A. M., Lubrini G., Madero-Jarabo R., Díez-Tejedor E., Fuentes B. (2019). Melodic intonation therapy in post-stroke nonfluent aphasia: a randomized pilot trial. *Clinical Rehabilitation*.

[6] Wang H., Li S. Q., Zhou Z. X., Yanhong D., Qingwei YU., Junjie LI. (2019). Damage to the dominant arcuate fasciculus degrades auditory comprehension in non-fluent aphasia. *Chin J Physical MedRehabil*.

[7] Litjens G., Kooi T., Bejnordi B. E. (2017). A survey on deep learning in medical image analysis. *Medical Image Analysis*.

[8] Wang G., Zuluaga M. A., Li W. (2019). DeepIGeoS: A Deep Interactive Geodesic Framework for medical image segmentation. *IEEE Transactions on Pattern Analysis and Machine Intelligence*.

[9] Criminisi A., Sharp T., Blake A. (2008). GeoS: Geodesic image segmentation. *Lecture Notes in Computer Science*.

[10] Lawes I. N. C., Barrick T. R., Murugam V. (2008). Atlas-based segmentation of white matter tracts of the human brain using diffusion tensor tractography and comparison with classical dissection. *NeuroImage*.

[11] Catani M., Dell’Acqua F., Vergani F. (2012). Short frontal lobe connections of the human brain. *Cortex*.

[12] Tae W. S., Ham B. J., Pyun S. B., Kang S. H., Kim B. J. (2018). Current clinical applications of diffusion-tensor imaging in neurological disorders. *Journal of Clinical Neurology*.

[13] Hosomi A., Nagakane Y., Yamada K. (2009). Assessment of arcuate fasciculus with diffusion-tensor tractography may predict the prognosis of aphasia in patients with left middle cerebral artery infarcts. *Neuroradiology*.

